# Supplementary material for: EnzML: multi-label prediction of enzyme classes using InterPro signatures
Source: BMC Bioinformatics. 2012 Apr 25;13:61. doi: 10.1186/1471-2105-13-61 (PMC3483700; doi:10.1186/1471-2105-13-61)
Supplement: Addtional file 5 — The Java code to format the data files, evaluate and predict. The file enzml_java_code.tar.gz contains the Java code used to format database data to ARFF and XML formats, to execute cross and train-test (jackknife) evaluations and to record evaluation results to database. More information is included in the readme.txt file and the Javadoc files. The code can be used with a MySQL database. To use a different database software, other JDBC drivers might be required. [file 1471-2105-13-61-S5.gz › java_code/enzml2011/doc/uk/ac/ed/inf/taweka/class-use/ArffGenerator.html]

Uses of Class uk.ac.ed.inf.taweka.ArffGenerator


---


|  |  |  |
| --- | --- | --- |
| **Overview** \| **Package** \| **Class** \| **Use** \| **Tree** \| **Deprecated** \| **Index** \| **Help** | |  |
| PREV   NEXT | **FRAMES**    **NO FRAMES**     **All Classes** |  |


---


## **Uses of Class uk.ac.ed.inf.taweka.ArffGenerator**

| Packages that use ArffGenerator | |
| --- | --- |
| **uk.ac.ed.inf.taweka** |  |
| **uk.ac.ed.inf.taweka.mulan** |  |
| **uk.ac.ed.inf.taweka.test** |  |

Uses of ArffGenerator in uk.ac.ed.inf.taweka

| Methods in uk.ac.ed.inf.taweka that return ArffGenerator | |
| --- | --- |
| `ArffGenerator` | `DataSetManager.getArffGenerator()` |
| `ArffGenerator` | `ArffPropsTableManager.getArffGenerator()` |

Constructors in uk.ac.ed.inf.taweka with parameters of type ArffGenerator

`ArffPropsTableManager(ArffGenerator arffGenerator)`
  
          Constructor 1: initialises the table manager to point to the arff table.

`DataSetManager(ArffGenerator generator)`

Uses of ArffGenerator in uk.ac.ed.inf.taweka.mulan

| Subclasses of ArffGenerator in uk.ac.ed.inf.taweka.mulan | |
| --- | --- |
| `class` | `MulanArffGenerator`             Generates an Attribute Relation Format File (ARFF) in Mulan format (multiple class labels per instance). |

Uses of ArffGenerator in uk.ac.ed.inf.taweka.test

| Methods in uk.ac.ed.inf.taweka.test that return ArffGenerator | |
| --- | --- |
| `static ArffGenerator` | `ArffGeneratorTest.getArffGenerator()` |
| `static ArffGenerator` | `ArffGeneratorTest.getArffGeneratorWithNoXmlProperty()` |

---


|  |  |  |
| --- | --- | --- |
| **Overview** \| **Package** \| **Class** \| **Use** \| **Tree** \| **Deprecated** \| **Index** \| **Help** | |  |
| PREV   NEXT | **FRAMES**    **NO FRAMES**     **All Classes** |  |


---
